# Sensor Data Fusion for Body State Estimation in a Bipedal Robot and Its Feedback Control Application for Stable Walking

**DOI:** 10.3390/s150304925

**Published:** 2015-02-27

**Authors:** Ching-Pei Chen, Jing-Yi Chen, Chun-Kai Huang, Jau-Ching Lu, Pei-Chun Lin

**Affiliations:** Department of Mechanical Engineering, National Taiwan University, No. 1, Sec. 4, Roosevelt Road, Taipei 10617, Taiwan; E-Mails: r00522810@ntu.edu.tw (C.-P.C.); luckyskyer@gmail.com (J.-Y.C.); ckhuang911@ntu.edu.tw (C.-K.H.); b93502114@ntu.edu.tw (J.-C.L.)

**Keywords:** body state estimator, sensor fusion, extended Kalman filter, bipedal robot, ZMP, preview control

## Abstract

We report on a sensor data fusion algorithm via an extended Kalman filter for estimating the spatial motion of a bipedal robot. Through fusing the sensory information from joint encoders, a 6-axis inertial measurement unit and a 2-axis inclinometer, the robot’s body state at a specific fixed position can be yielded. This position is also equal to the CoM when the robot is in the standing posture suggested by the detailed CAD model of the robot. In addition, this body state is further utilized to provide sensory information for feedback control on a bipedal robot with walking gait. The overall control strategy includes the proposed body state estimator as well as the damping controller, which regulates the body position state of the robot in real-time based on instant and historical position tracking errors. Moreover, a posture corrector for reducing unwanted torque during motion is addressed. The body state estimator and the feedback control structure are implemented in a child-size bipedal robot and the performance is experimentally evaluated.

## 1. Introduction

Due to the rapid growth in robotics in recent decades, it is unavoidable that robots should come into our lives, interacting with us and the environment. From this point of view, robots’ perception plays a key role, and it is sensory information, which relays messages from outside of the robot, that makes it possible for robots to perceive [1]. In order to improve the capability and reliability of the sensory information from various modalities, they can be fused [2,3]. In the field of mobile robots, the concept of sensor data fusion has already been widely used on wheeled vehicles. Several practical applications have been addressed, including fusion between an inertial measurement unit (IMU) and vision [4], Kinect™/laser fusion [5], odometry/laser fusion via extended Kalman filter (EKF) for robotic guidance [6], laser/GPS fusion via Kalman filter for tracking in outdoor environments, laser/vision fusion for human detection [7], and Kinect™/thermal fusion for robust people detection. Moreover, multi-sensor information was employed in the European URUS (Ubiquitous Networking Robotics in Urban Sites) project, which aims to develop a network of robots to collaborate and communicate with human beings and the environment in urban areas [8].

On the other hand, in the field of legged robots, which has focused on motion development and stability improvement in the past, there have been only limited studies on utilizing sensor fusion to stabilize robot behavior. However, due to the rapid development of modern legged robots, the underlying principle for creating stable locomotion has been revealed. As a result, more and more research concerning legged robots has focused on how to enhance the robot’s performance through sensory feedback algorithms and data fusion. For example, a velocity estimation method based on the kinematics model and IMU for a bipedal robot was addressed [9]. A full body state estimation algorithm was proposed based on IMU and legged odometry for a quadruped [10]. Both of these employed the EKF. A vision-based obstacle avoidance navigation system for humanoid robots has also been reported [11]. A pose estimation methodology was developed for a six-legged walking robot, which fuses the sensory information from IMU, vision and leg odometry [12]. By fusing the sensory data from IMU, electric compass and touch sensors, a state estimation algorithm can be established for a hexapod robot to accomplish a two-dimensional navigation task [13]. Regarding humanoid robot research, an observer-based approach is utilized for stabilizing bipedal walking motion based on the 3-dimensional linear inverted pendulum model (LIPM) and preview control [14]. In addition, an EKF-based state estimation method is introduced using only common proprioceptive sensors and leg kinematics [15]. In our previous research, a leg-strain-based configuration estimator was developed for a hexapod robot [16]; later, the sensor was fused with an IMU via EKF for estimating the full body state of a hexapod robot [17]. An IMU/encoder fusion model-based feedback control was also developed for improving the stability of the robot’s jogging motion [18]. In addition, a 9-axis IMU [19] and a 12-axis gyroscope-free IMU [20] were developed for better motion state reconstruction.

As the environments are already designed for humans, humanoid robots in comparison to other types of robots have a unique and incomparable importance to human society. To successfully build a humanoid robot and achieve human locomotion in machine form, creating stable walking must play a key role, and other behaviors, such as turning [21,22] and running [23], must also be taken into account. With the well-developed locomotion strategy based on the concept of Zero-Moment Point (ZMP) [24], the walking controller of the bipedal robots can be composed of two stages: generation of a stable walking pattern in the sense of ZMP, and a real-time control strategy that tracks the robot’s ZMP to the planned value. However, practically, the discrepancy between the theoretical model and the empirical robot, such as the backlash in mechanism, un-modeled dynamics, and disturbance of the environment, all affect the stability of the locomotion. To improve the performance of robotic motion, many studies regarding stable walking pattern generation have been reported [25,26,27]. In these approaches, the robots are often simplified as a point mass located at its center of mass (CoM) and moved as an inverted pendulum. By specifying the dynamic relations between the CoM and the ZMP, the CoM trajectory corresponding to the pre-planned ZMP trajectory can be generated. In order to grant walking stability to the bipedal robots, the implementation of a real-time feedback controller is crucial to deal with the uncertainty and disturbance from the outside, and the controller usually relies on the sensory information of the body state or ground reaction forces. Nishiwaki *et al.* regulated the CoM position of the robot according to ZMP errors [28]. Sato *et al.* developed a ZMP disturbance observer to realize the position and acceleration compensation of the CoM [29]. Buschmann *et al.* proposed another effective approach, which used position/force control [30]. Stephens *et al.* proposed a dynamic balance force control, which considers the full rigid-body dynamics of the robot to produce the desired contact force [31]. Most of these methods require force measurement between the foot and the ground.

In our previous work on the empirical bipedal robot, the robot successfully fulfilled some tasks, such as walking and turning, using a preview-control-based open-loop control strategy [32]. However, instability due to the backslash of the mechanism as well as unmodeled dynamics encouraged us to develop a multi-sensor feedback control strategy to improve the stability of the locomotion [33]. As a result, we investigated the feasibility of stabilizing bipedal walking by sensory feedback using the state of the body at a specific position, which is hereafter referred to as the “FP.” This position is also equal to the CoM when the robot is in the standing posture suggested by the detailed CAD model of the robot. A body state estimator based on the LIPM was designed, fusing the sensory information of joint angles (from encoders), body acceleration and angular velocity (from a 6-axis IMU), and body inclination (from an inclinometer) via an extended Kalman filter. Then, a walking stabilizer based on the estimated body position (*i.e.*, FP) was designed. Furthermore, a posture corrector using the information from the 6-axis force sensors on the ankles was introduced to eliminate unwanted torque during motion. Note that Kajita *et al.* also designed a body posture controller to regulate the CoM and a ground contact wrench based on the simple inverted pendulum model [34]. His work focused on controlling the robot’s walking on surfaces at the macro-scale (such as walking from an even flat surface to an inclined flat surface). In contrast, our work focuses on micro-scale regulation of the body posture. The novelty of this work lies in the design of the body state estimator, which combines the advantages of “leg odometry” and dynamic IMU-based state information. Following this, the estimated state is deployed for body motion regulation. Finally, the proposed control structure was implemented on a child-sized bipedal robot and experimentally evaluated.

The rest of this paper is organized as follows. Section 2 describes the design of the proposed body state estimator through multi-sensor data fusion. Section 3 reports the feedback control strategy to regulate and stabilize the walking bipedal robot based on the proposed body state estimator. Section 4 briefly introduces the empirical bipedal robot. Section 5 reports the experimental evaluation of the estimator and regulator, while Section 6 concludes the work.

## 2. The Body State Estimator

The method of generating a nominal CoM trajectory is based on the preview control strategy proposed by Kajita [35], which links a pre-planned ZMP trajectory ${p_{x}}^{ref}(k)$ to the CoM motion through the LIPM. Based on the preview control law, once the desired ZMP trajectory is defined, the CoM trajectory can be determined and controlled through the inverse kinematics of the robot using the sensory information from the joint encoders. However, because of the backlash in the mechanism and unmodeled dynamics of the system, the real CoM position is difficult to obtain using only the joint encoders. Consequently, the purpose of developing the multi-sensor-based body state estimator is to obtain a more precise body state and also to act as a reference for the feedback control strategy.

The body state estimator is a key component in this study, which estimated the state as located at a fixed position on the body (*i.e.*, FP). This position is also equal to the CoM when the robot is in the standing posture suggested by the detailed CAD model of the robot. Note that the CoM position varies while the robot walks, but the variation is less than 15 mm according to the CAD model. The state estimator can be further divided into two parts: the translational part and the rotational part. As described in the previous section, the proposed LIPM model-based control strategy only requires the CoM state (*i.e.*, translational only). However, in order to correctly estimate the CoM state, the complete body state estimator, including translation and orientation, is still required because the body orientation is necessary for transforming the raw strapdown IMU readings into those in the inertial frame. These two parts will be described separately below.

On the translational side, since motions in the three spatial directions are independent, the motion model can be decoupled into three discrete and linear KFs, each in charge of motion along with a specific principle axis in the inertial frame. An exemplary presentation of the translational KF model in the fore-aft direction is given below, where the constant acceleration model is utilized in the prediction stage:

(1) $${\hat{x}}_{k}^{t}=\left[\begin{array}{ccc} 1 & T & T^{2}/2\\ 0 & 1 & T\\ 0 & 0 & 1 \end{array}\right]{\hat{x}}_{k-1}^{t}+w_{k-1}^{t}\;{\tilde{z}}_{k}^{t}=\left[\begin{array}{c} \begin{array}{ccc} 1 & 0 & 0 \end{array}\\ \begin{array}{ccc} 0 & 0 & 1 \end{array} \end{array}\right]{\hat{x}}_{k}^{t}+v_{k}^{t}$$

where T is the sample period, and ${\hat{x}}^{t}\equiv {\left[\begin{array}{ccc} \hat{x} & \dot{\hat{x}} & \ddot{\hat{x}} \end{array}\right]}^{T}$ is the estimated translation state of the FP of the body, including position $\hat{x}$, velocity $\dot{\hat{x}}$, and acceleration $\ddot{\hat{x}}$. The symbols $w^{t}$ and $v^{t}$ represent the standard process and measurement noise variables given by covariance $Q^{t}$ and $R^{t}$, respectively, which are assumed to be independent and normally distributed with zero means. The measurement state ${\tilde{z}}^{t}\equiv \left[\begin{array}{cc} \tilde{x} & \ddot{\tilde{x}} \end{array}\right]$ includes position $\tilde{x}$ and acceleration $\ddot{\tilde{x}}$, and the derivation of these two states will be introduced separately in the following paragraphs.

The FP position measurement $\tilde{x}$ is computed based on the leg joint configuration [16], which means the FP position can be determined using the joint encoders according to the kinematics of the robot [32]. Since the empirical bipedal robot walks slowly and steadily, it is reasonable to assume that its foot contacts the ground rigidly without any slippage during locomotion. In each stride when the robot is supported by one leg, the robot body configuration with respect to the ground-contact foot can be computed according to the forward kinematic. When the robot is supported by both feet, the relative configuration between the two feet can also be derived based on legged kinematics. Thus, through sequential composition, the body configuration (*i.e.*, FP) of the robot at its jth stride relative to its initial configuration (*i.e.*, the “inertial frame”) at all instants can be computed. The quantitative computation can be represented as

(2) $$\left(\tilde{x}\left(j\right),\tilde{y}(j),G(j)\right)=FK(\tilde{\Theta }(j),G\left(j-1\right))$$

where G is landing position, and $\tilde{\Theta }$ is an array composed of leg joint angles, measured directly from motor encoders.

**Figure 1:**
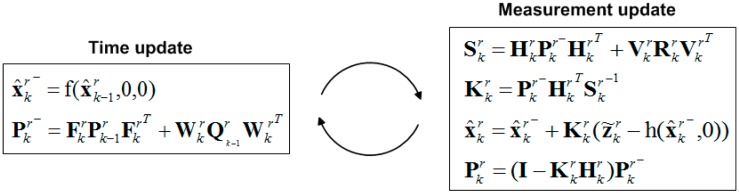
Flowchart of the quaternion-based extended Kalman filter for complete body state estimation.

On the rotational side, a quaternion-based extended Kalman filter is built to estimate body orientation, and the estimated state is used to perform gravity compensation and coordinate transformation of the accelerometer readings [17]. The model can be represented as

(3) $${\hat{x}}_{k}^{r}={\text{f}}^{r}({\hat{x}}_{k-1}^{r},0,w_{k-1}^{r})\;{\tilde{z}}_{k}^{r}={\text{h}}^{r}({\hat{x}}_{k}^{r},v_{k}^{r})$$

where the body state ${\hat{x}}^{r}\equiv (\hat{q},\hat{\omega })$ is composed of the body orientation represented by quaternion $\hat{q}={\left[q_{0}\;q_{1}\;q_{2}\;q_{3}\right]}^{T}$ and spatial angular velocity $\hat{\omega }$. The measurement state ${\tilde{z}}^{r}\equiv (\tilde{R},\tilde{P},\tilde{Y},\tilde{\omega })$ is composed of the body orientation represented in pitch, roll, and yaw, and spatial angular velocity $\tilde{\omega }=[w_{x}\;w_{y}\;w_{z}]$. The symbols $w^{r}$ and $v^{r}$ represent the standard process and measurement noise variables given by covariance $Q^{r}$ and $R^{r}$, respectively. Both ${\text{f}}^{r}$ and $h^{r}$ are non-linear functions, where ${\text{f}}^{r}$ is the relation between the previous state and current state under the assumption of constant angular speed motion, and $h^{r}$ projects the orientation represented in orientation form onto the measurement body orientation represented in pitch, roll, and yaw, and spatial angular velocity. The flowchart of the quaternion-based extended Kalman filter is depicted in Figure 1.

The non-linear functions ${\text{f}}^{r}$ and $h^{r}$, as well as the linearized matrix $F^{r}$ and $H^{r}$, are shown below:

(4) $$\begin{array}{cc} {{\hat{x}}_{k}^{r}}^{-}={\left[\begin{array}{c} \hat{q}\\ \hat{\omega } \end{array}\right]}_{k}={\left[\begin{array}{ccccccc} \text{cos}\frac{\Delta \theta }{2} & 0 & 0 & 0 & -q_{1}\alpha & -q_{2}\alpha & -q_{3}\alpha \\ 0 & \text{cos}\frac{\text{Δ}\theta }{2} & 0 & 0 & q_{0}\alpha & -q_{3}\alpha & q_{2}\alpha \\ 0 & 0 & \text{cos}\frac{\text{Δ}\theta }{2} & 0 & q_{3}\alpha & q_{0}\alpha & q_{1}\alpha \\ 0 & 0 & 0 & \text{cos}\frac{\text{Δ}\theta }{2} & -q_{2}\alpha & q_{1}\alpha & q_{0}\alpha \\ 0 & 0 & 0 & 0 & 1 & 0 & 0\\ 0 & 0 & 0 & 0 & 0 & 1 & 0\\ 0 & 0 & 0 & 0 & 0 & 0 & 1 \end{array}\right]}_{k-1}{\left[\begin{array}{c} q_{0}\\ q_{1}\\ q_{2}\\ q_{3}\\ \omega_{x}\\ \omega_{y}\\ \omega_{z} \end{array}\right]}_{k-1}\\ x_{k}^{r}=F_{k}^{r}x_{k-1}^{r}\\ h^{r}\left({\hat{x}}^{r},0\right)={\left[\begin{array}{cccccc} \tilde{R} & \tilde{P} & \tilde{Y} & \omega_{x} & \omega_{y} & \omega_{z} \end{array}\right]}^{T}\\ ={\left[\begin{array}{cccccc} -{\text{tan}}^{-1}\frac{R_{23}}{R_{33}} & {\text{tan}}^{-1}\frac{R_{13}}{R_{33}} & {\text{tan}}^{-1}\frac{R_{21}}{R_{11}} & \omega_{x} & \omega_{y} & \omega_{z} \end{array}\right]}^{T} & H_{\left[i,j\right]}^{r}=\frac{\partial h{\left(x^{r}\right)}_{\left[i\right]}}{\partial x_{\left[j\right]}}\\ =\left[\begin{array}{ccccccc} H_{11} & H_{12} & H_{13} & H_{14} & 0 & 0 & 0\\ H_{21} & H_{22} & H_{23} & H_{24} & 0 & 0 & 0\\ H_{31} & H_{32} & H_{33} & H_{34} & 0 & 0 & 0\\ 0 & 0 & 0 & 0 & 1 & 0 & 0\\ 0 & 0 & 0 & 0 & 0 & 1 & 0\\ 0 & 0 & 0 & 0 & 0 & 0 & 1 \end{array}\right] \end{array}$$

where $F^{r}$ is the linearized system matrix, $H^{r}$ is the linearized matrix of $h^{r}$. Here, we set $\text{α}=\text{sin}\frac{\text{Δθ}}{2}\frac{\text{Δ}t}{\text{Δθ}}$ and $\hat{q}=q_{0}+q_{1}i+q_{2}j+q_{3}k=cos\frac{\text{θ}}{2}+sin\frac{\text{θ}}{2}\hat{n}$, where θ represent the rotation angle and the unit vector $\hat{n}$ indicates the axis of rotation. Refer to the Appendix for the components of the matrix H. The details of the quaternion computation can be found in [36].

Considering the feedback sensors, a 2-axis inclinometer is used to measure roll and pitch $\left(\tilde{\text{R}},\tilde{\text{P}}\right)$, and a 3-axis gyro in the IMU is used to obtain angular velocity $\tilde{\omega }$. Although the inclinometer has a similar internal structure to the accelerometer, and it indeed uses the gravity vector as the criterion for inclination measurement, the commercial inclinometer is utilized owing to its precision and low noise level. When the robot performs straight walking (*i.e.*, no yaw motion involved), the yaw measurement $\tilde{\text{Y}}$ is set to zero in the empirical implementation. When the work extends to the turning motion, the yaw orientation of the robot was set to be the nominal trajectory. With estimated body orientation $\hat{q}$ by Equation (3) and measured linear acceleration in the body frame $\tilde{a}$ by the 3-axis accelerometer in the IMU, the body linear acceleration in the inertial frame can be derived through coordination transformation and after gravity compensation:

(5) $$(\ddot{\tilde{x}},\ddot{\tilde{y}})=W(\hat{q},\tilde{a})$$

which is used as the measured acceleration in Equation (1). Note that the world coordinate system is set at the beginning of the walk, and the relationship between the body and the world coordinate system in the legged odometry is established by the sequential composition of joint encoder data and leg kinematics. The legged odometry data is further fused with the EKF to estimate the body state.

It is necessary to mention here that the CoM moves during robot locomotion, and this phenomenon occurs on all kinds of multi-legged platforms because the legs are, in general, not massless. The IMU is fixedly mounted on the robot (*i.e.*, a strapdown IMU) at the position equivalent to the robot CoM when the robot is in standing posture. The complete CAD model of the robot reveals that the CoM varies by less than 15 mm in our walking experiment. When the CoM moves during a walking experiment (the position can be correctly estimated by the joint angles), its translational state can be computed from the IMU translational state plus the effect of angular acceleration (*i.e.*, can be computed kinematically by the 12-axis IMU mount on the robot [20]) and angular velocity (*i.e.*, by the gyro). Because the robot body is programmed to move without orientation variation during walking, the angular state is comparably small. We found that the acceleration difference between the CoM and the IMU is not significant. In addition, we found that the acceleration computation that occurs as a consequence of this position shifting is noisy because, in our case, the distance between the two positions is small and the angular motion is not obvious. As a result, we found that the state difference between the FP and actual CoM in our application was small and decide to use the estimation at the FP as the reference position for feedback, rather than to precisely estimate the CoM position in our current imprecisely built robot.

**Figure 2:**
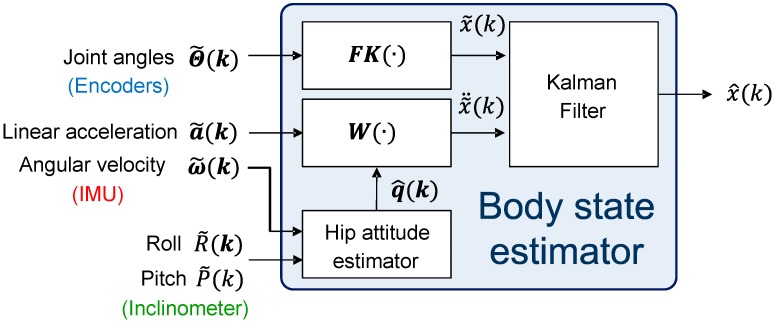
Structure of the body state estimator.

To offer a brief summary of this section, we intend to use the body state but not the ZMP state as the sensory information for feedback control. Therefore, creating a robust body state estimator is an important task that must be completed before the implementation of the control strategy. The idea of sensor fusion is to combine the advantages of “leg odometry” and dynamic IMU-based state information. For the bipedal walking robot with foot contact, the sequential composition of the forward/inverse kinematics with joint encoders would ideally be able to deliver non-drift body pose information. However, the computation on the physical robot is compromised by imperfect empirical conditions such as backlash, assembly inaccuracy, rigidity of the structure, *etc.* One obvious example is that when the robot is programmed to pose in double stance according to kinematics, there is a foot positioning error on the physical robot of up to 2 cm, so that one of the feet is not able to flatly contact the ground. In addition, when the foot slips, the composition has some drift error, and this is the reason we called it “legged odometry.” In contrast to the encoders which provide information on the position state, the IMU provides the derivative state or the double derivative state, which captures the dynamics of the robot better. However, the IMU-only system is not good for position or orientation estimation (*i.e.*, drift problem) because of unobservability. As a result, the body state can be better estimated by fusing both encoder- and IMU data. For each DOF, there are two sensory inputs for fusion. Translational DOFs use encoders and accelerometers. Pitch and roll use inclinometers and gyros. Ideally, the bipedal kinematics based on encoder data can yield body orientation as well, and this was indeed our first method for yielding orientation for fusion. However, the encoders’ orientation estimation on the physical robot was very bad (about 3 degrees), resulting from imperfect empirical conditions such as backlash, assembly inaccuracy, rigidity of the structure, *etc.* Therefore, the inclinometer was utilized, which provided better estimation on the robot. Though the inclinometer is not good in a dynamic environment because it is an accelerometer-based device, the sensing accuracy is tolerable for our current walking experiment. Furthermore, the raw data coming from each sensor (encoders and accelerometers or inclinometer and gyroscopes) are not correctly related through derivation because both sensors are noisy. This is the major reason why we use the (extended) Kalman filter, which fuses the data and provides the correctly related estimation. Finally, Figure 2 shows the overall information flow of the proposed body state estimator.

## 3. Multi-Sensor Feedback Control Algorithm for the Stable Walking of a Bipedal Robot

Theoretically, by utilizing the nominal trajectory according to the preview control law, the corresponding CoM trajectory can be derived based on the preplanned ZMP trajectory. Then, the position of each joint angle at each point in time can be obtained via inverse kinematics of the robot. However, in reality, the empirical CoM trajectory of the robot does not follow the planned version owing to many uncertainties, such as backlash in the mechanism, un-modeled dynamics, disturbance, discrepancy between robot and LIPM, *etc.* To remedy this unwanted phenomenon, a real-time controller is implemented to regulate the actual body trajectory of the robot to approach the nominal one and further improve stability while the robot is in motion. This controller relies on multi-sensor information, including joint angles (from encoders), acceleration (from IMU), angular velocity (from IMU), and force and torque of the feet (from force sensors). This multi-sensor feedback controller is composed of two parts: a body state feedback control algorithm and a foot posture corrector. These two parts are described separately below.

### 3.1. Body State Feedback Control Algorithm

In the proposed body state feedback control, the state of the fixed position (*i.e.*, FP) estimated by the body state estimator was employed to modify the difference between the current FP position and the nominal one. Since the displacement modification is directly added to the FP position, a damping controller is chosen to smooth position modification [34]. While a PID controller was also tried, it caused chattering in simulations and preliminary experiments. By utilizing the estimator that provides the empirical FP trajectory $\left(\hat{x}(k),\hat{y}(k)\right)$ described in Section 2 and the computed nominal FP trajectory (x(k),y(k)) described in Equation (2), the adjustment of FP displacement can be set according to the following:

(6) $$\text{Δ}\dot{x}(k)=k_{d}\left(x\left(k\right)-\hat{x}\left(k\right)\right)-\frac{1}{T_{d}}\text{Δ}x(k)$$

The damping controller can be separated into two parts. The first part is a position controller (*i.e.*, $k_{d}\left(x\left(k\right)-\hat{x}\left(k\right)\right)$), where x(k) represents the nominal FP trajectory derived based on the preview control law, $\hat{x}\left(k\right)$ represents the estimated FP position, and $k_{d}$ is the proportional gain. The second part functions as a damper to smoothen the response of the controller, where the variation of the position (*i.e.*, Δx(k)) can be numerically derived through the first-order differential equation. Thus, $T_{d}$ is a time constant. Finally, the calculated Δx(k) is added to the preview controller by equation x(k+1)=x(k)+Δx(k) to compensate for the discrepancy between the estimated FP position and nominal FP position. Thus, if the gain $k_{d}$ is small, the effect of the real-time adjustment is minor and the robot would move according to its nominal motion. In contrast, if $k_{d}$ is too large, the compensation may overcorrect the robot and the robot may unstably overshoot in the compensated direction. If $T_{d}$ is small, the effect of modification decreases quickly. On the other hand, if $T_{d}$ is large, it causes a slow response and the robot may again unstably move toward the over-compensated direction. With the control strategy described in Equation (6), the position error would be regulated to improve the walking performance of the robot. Note that the strategy of regulating the FP trajectory along with the lateral y-direction is implemented independently and in the same manner.

### 3.2. Foot Posture Corrector

The ZMP position of the robot’s motion can be determined by the CoM position and CoM acceleration based on the preview control. Nevertheless, the true ZMP during the motion depends on the complex reaction of force and torque while the foot of the robot contacts the ground. In order to obtain the real ZMP position during the motion, the 6-axis force sensors mounted on the ankles of the robot were utilized. The real ZMP position of each foot can be determined by the following equation:

(7) $${\text{P}}_{ZMPx,i}=\frac{P_{x,i}F_{z,i}-P_{z,i}F_{x,i}-M_{y,i}}{F_{z,i}}\;{\text{P}}_{ZMPy,i}=\frac{P_{y,i}F_{z,i}-P_{z,i}F_{y,i}-M_{x,i}}{F_{z,i}}\;(i=L,R)$$

where P represents the position of the force sensors, and F and M represent the force and moment measured from the force sensors. R and L indicate the left foot and right foot respectively.

**Figure 3:**
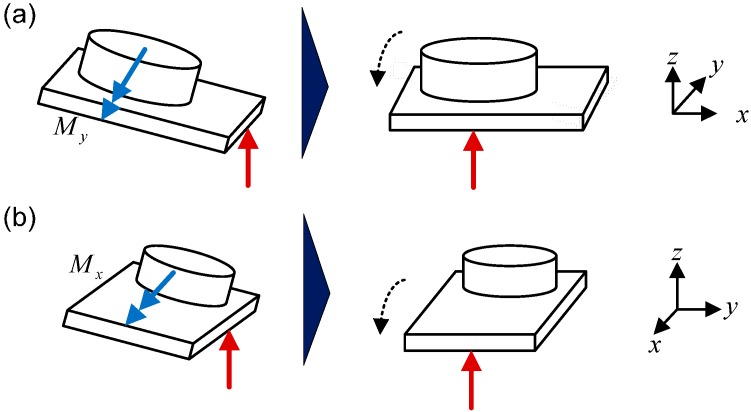
Schematics of the (**a**) pitch adjustment and (**b**) roll adjustment of the robot.

Considering the control strategy, the main disturbance is the unwanted torque, which results in the bumpy contact with the ground as shown in Figure 3. This situation conflicts with the assumption in the LIPM model utilized in preview control that the sole of the foot contacts the ground horizontally. Consequently, a torque control strategy was developed for rejecting this extra torque. As can be seen in the schematic drawing in Figure 3a, an external negative torque $M_{y}$ will cause the ZMP position to shift to positive x-direction. Thus, the foot posture corrector was developed to amend this error by adding an additional pitch angle to the ankle. Similarly, while an external positive torque $M_{x}$ causes the ZMP position to shift to positive y-direction, this corrector will adjust the roll angle of the ankle.

Before adjusting the angle of the ankle, the reference force profile should be calculated by using the ZMP distributor [34]. Thus, the measured force data can be compared with the reference data. The LIPM provides the overall force information while the force sensors mounted on each foot give the force of each leg. Therefore, the force mapping that distributes the total force to each leg is:

(8) $${\text{F}}_{L}^{d}=-\left(1-\text{α}\right)\text{mg}\;{\text{F}}_{R}^{d}=-\text{αmg}\;{\text{M}}_{i}^{d}=\left({\text{P}}_{i}-P_{ZMP}^{d}\right)\times {\text{F}}_{i}^{d}\;(i=L,R)$$

where

$$\text{α}=\frac{\left|{\text{P}}_{ZMP}^{d}-{\text{P}}_{L}\right|}{\left|{\text{P}}_{L}-{\text{P}}_{R}\right|}$$

**Figure 4:**
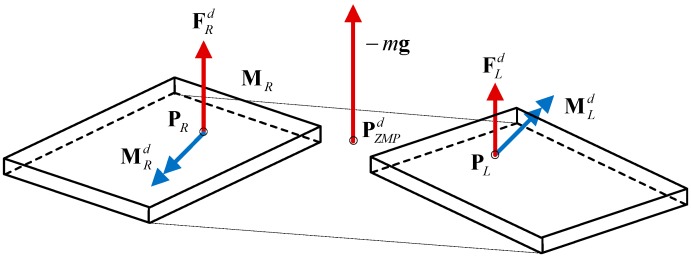
Forces and moments of the bipedal robot.

${\text{P}}_{ZMP}^{d}$, $F_{i}^{d}$ and $M_{i}^{d}$ represent the nominal ZMP position, force and torque, respectively. ${\text{P}}_{i}$ represents the position of the ankle projecting on the foot. R and L indicate the left foot and right foot respectively. Note that α=1 indicates that the ZMP position is on the right foot, while α=0 indicates that the ZMP position is on the left foot. The symbols m and g represent the mass of the robot and gravity constant respectively. Here, the force distribution is simply designed based on the linear weight ratio of the ground reaction forces applied to two feet. If the acceleration of the CoM is not obvious (*i.e.*, the robot moves in a quasi-static manner), the equation can be derived based on a force equation in static equilibrium. The precise force distribution between two legs may not be crucial because the supporting area formed by the two legs (*i.e.*, in double stance phase) is much larger in comparison to the single foot area (in single stance phase). Figure 4 illustrates the force diagram mentioned above.

Moreover, the equation below transfers the objective torque on the world frame to the frame of the ankle joints.

(9) $${{\text{M}}^{\prime }}_{i}^{d}=\left[\begin{array}{c} {{\text{M}}^{\prime }}_{i,x}^{d}\\ {{\text{M}}^{\prime }}_{i,y}^{d}\\ {{\text{M}}^{\prime }}_{i,z}^{d} \end{array}\right]=\left[\begin{array}{ccc} \text{cos}(-{\text{θ}}_{i}) & -\text{sin}(-{\text{θ}}_{i}) & 0\\ \text{sin}(-{\text{θ}}_{i}) & \text{cos}(-{\text{θ}}_{i}) & 0\\ 0 & 0 & 1 \end{array}\right]\left[\begin{array}{c} {\text{M}}_{i,x}^{d}\\ {\text{M}}_{i,y}^{d}\\ {\text{M}}_{i,z}^{d} \end{array}\right]\;\left(\text{i}=\text{L},\text{R}\right)$$

where ${\text{M}}^{d}$ and ${\text{M}}^{\prime d}$ represents the objective torque on the world frame and the objective torque of the frame of the ankle joints. R and L indicate the left foot and right foot respectively. Finally, the torque controller can be designed by using the damping controller previously mentioned.

(10) $$\text{Δ}{\dot{\text{θ}}}_{i,roll}=k_{d,torque}\left({\tilde{M}}_{i,x}-{{\text{M}}^{\prime }}_{i,x}^{d}\right)-\frac{1}{T_{d,torque}}\text{Δ}{\text{θ}}_{i,roll}\;(i=L,R)\;-\text{Δ}{\dot{\text{θ}}}_{i,pitch}=k_{d,torque}\left({\tilde{M}}_{i,y}-{{\text{M}}^{\prime }}_{i,y}^{d}\right)-\frac{1}{T_{d,torque}}\text{Δ}{\text{θ}}_{i,pitch}\;(i=L,R)$$

where $k_{d,torque}$ is a position feedback gain, $T_{d,torque}$ is a time constant, and $\tilde{M}$ is the measured torque from the force sensors. By appropriately choosing the parameters, the corrector can compensate for the ZMP position error in order to improve the walking stability of the robot. To sum up, the proposed foot posture corrector is mainly used to correct the tilted foot contact with the ground resulting from the imperfect empirical robot conditions, not to globally alter the body motion.

**Figure 5:**
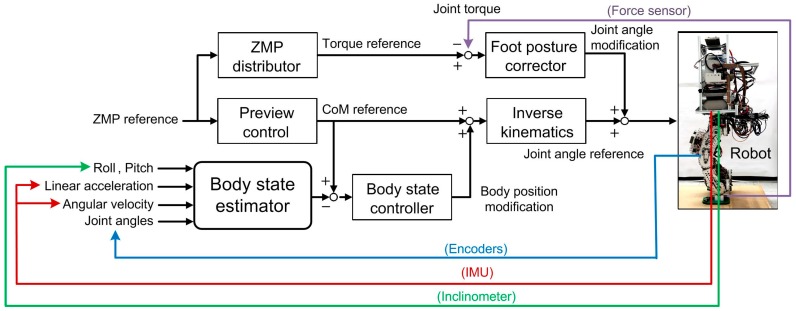
Structure of the multi-sensor feedback controller, including the body state feedback controller and foot posture corrector.

Considering the capability of each controller, the body state feedback controller eliminates the difference between the nominal and robot body trajectories while the foot posture corrector is mainly used to correct the tilted foot contact with the ground, not to globally alter the body motion. Therefore, the foot posture corrector has a fast response, so when the foot contacts the ground, its imperfect tilt angle can be quickly corrected. In contrast, the body state feedback controller is designed to have relatively small gain, compared with the foot posture corrector, in order to reduce the level of unwanted acceleration in the system. Large gain may not change the body motion but would upset the foot posture owing to the greater inertia of the body. Finally, Figure 5 shows the block diagrams of the proposed multi-sensor feedback controller for stable bipedal walking, including the body state feedback control algorithm and foot posture corrector.

To give a brief summary of this section, the motivation for the proposed control strategy is to check whether the body-only feedback is sufficient for stable bipedal walking. The main research stream focuses on the ZMP-based control strategy or its use with other strategies, and this approach indeed addresses the stability of the robot better. Here, our concept is mainly based on the approach of general multi-legged systems where CoM motion is the major concern for locomotion control.

## 4. The Bipedal Robot and Experiment Setup

**Figure 6:**
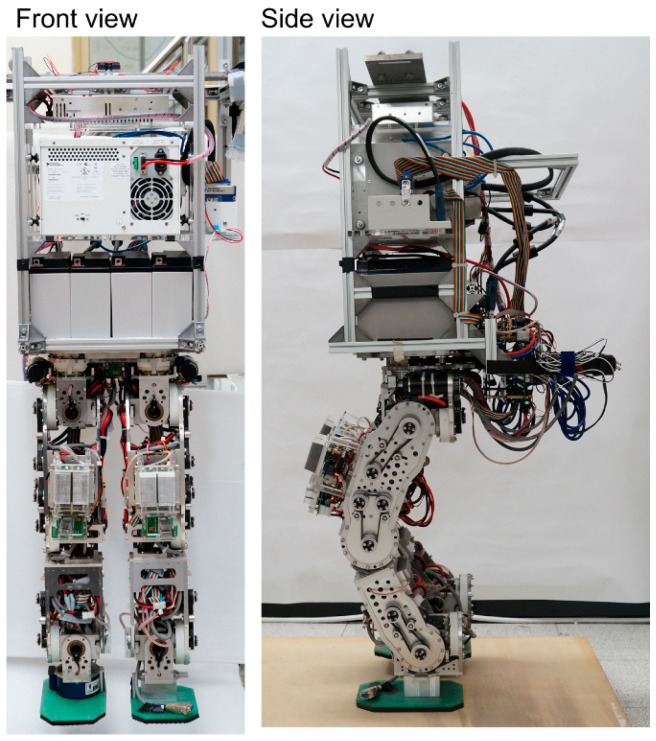
Picture of the child-sized bipedal robot that serves as the platform for experimental evaluation.

The child-sized bipedal robot shown in Figure 6 is utilized for experimental evaluation of the proposed body state estimator and the multi-sensor feedback control algorithm. The robot is 1.27 m in height and 78 kg in mass, and it has 6 active degrees of freedom in each leg. A real-time (RT) embedded control system is installed on the robot as the main computation power, including a PXI chassis (PXI-1031DC, National Instruments (NI), Austin, TX, USA), a PXI embedded controller (PXI-8110, NI), a digital I/O board with programmable FPGA (PXI-7813R, NI), and an expansion chassis (NI 9151, NI) with an A/O module (NI 9264, NI) and an A/I module (NI 9205, NI). All active joints are driven by brushed DC motors (RE-40, Maxon, Sachseln, Switzerland) with integrated magnetic encoders for joint angle measurement. The robot has an IMU comprising one 3-axis accelerometer (ADXL330, Analog Device, Norwood, MA, USA) and three 1-axis rate gyros (ADXRS610, Analog Device) mounted at a fixed point on the body, which is also equal to the CoM when the robot is in the standing posture. It also has a 2-axis inclinometer (SCA100T, VTI Instrument, Irvine, CA, USA) for body inclination measurement. In addition, the robot has two 6-axis force/torque transducers (IFS-90M40A100-I50-ANA, NITTA, Suwanee, GA, USA) mounted on the ankles. The software in the RT processor and the FPGA run with 1 kHz and 10 kHz sampling rates, respectively.

**Figure 7:**
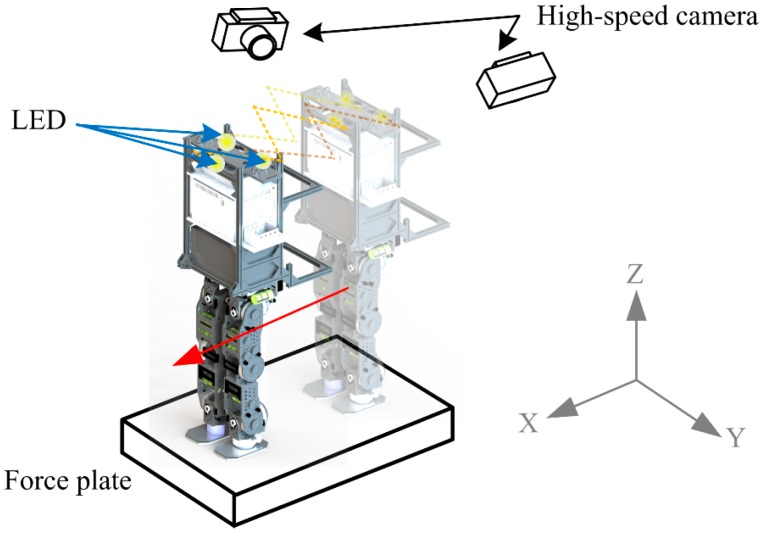
Experimental setup for bipedal walking.

In order to quantitatively evaluate the performance of the estimator and the controller, several experiments were executed whereby the robot ran within the ground truth measurement system (GTMS) as shown in Figure 7, which is composed of two 500 Hz high-speed cameras (A504k, Basler, Ahrensburg, Germany) and one 6-axis force plate (FP4060-07, Bertec, Columbus, OH, USA). The high-speed cameras are mounted on the top right and left sides of the runway. When a bright marker is captured by both cameras, its spatial coordinate can be reconstructed. By installing three bright LEDs on top of the robot, the spatial 6-DOF body state can be derived according to the geometrical relation between the markers and the robot’s fixed body position. The 6-axis force plate is mounted in the middle of the runway to capture the force interaction between the ground and the robot. More details can be found here [37].

## 5. Experimental Results and Discussion

### 5.1. Evaluation of the Proposed Body State Estimator

**Figure 8:**
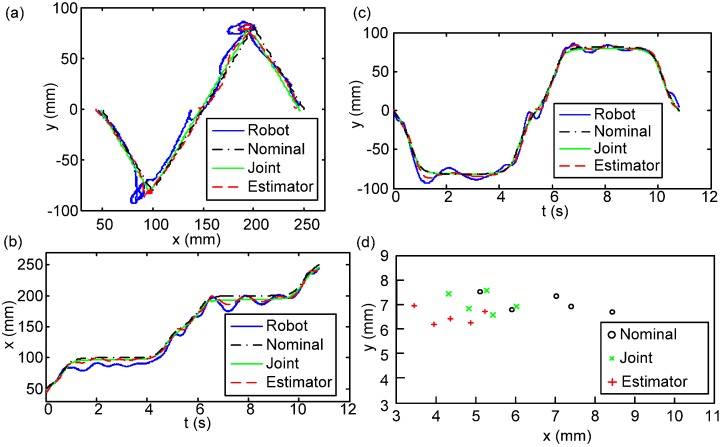
The robot body trajectories (*i.e.*, FP) computed according to different methods: Nominal trajectory (black dash-dotted), empirical robot trajectory (blue solid), joint trajectory (green solid), and estimated trajectory (red dashed). (**a**) Trajectories plotted in the xy-plane; (**b**) Fore-aft trajectory (x) *vs.* time; (**c**) Lateral trajectory (y) *vs.* time; (**d**) RMS errors of all runs.

In these experiments, the robot started from a standing state then walked 30 cm at 0.1 m/s on the force plate and returned to the standing posture. The durations of the single-leg support phase and double-leg support phase are 3.6 s and 1.8 s, respectively. To evaluate the performance of the body state estimator, the robot walked within the GTMS 5 times and its leg joints were operated according to the nominal CoM trajectory generated by the preview control. One of the experimental results is plotted in Figure 8a–c as the exemplary presentation, where Figure 8a shows the planar trajectory from the top view and Figure 8b,c plot the displacement *vs.* time in the fore-aft (x) and lateral (y) directions. Four FP trajectories are plotted in Figure 8a–c: (I) the actual FP trajectory of the robot captured by GTMS (referred to as “robot trajectory”); (II) the planned FP trajectory computed by the preview controller (*i.e.*, nominal trajectory); (III) the FP trajectory computed by forward kinematics with empirical joint-angle inputs measured by encoders (referred to as “joint trajectory”); and (IV) the estimated FP trajectory by the proposed estimator (referred to as “estimated trajectory”). Figure 8a–c reveals that the joint trajectory is almost identical to the nominal trajectory, which means the joint position tracking on the robot can keep the relative relation between the landing position and FP. However, because this method cannot reflect the imperfections of the empirical system such as mechanism backlash or small-swing caused by un-rigid deformation on rubber foot soles and legged links or joints, the empirical FP trajectory of the robot is not the same as the nominal one. On the other hand, with the addition of the IMU that captures the actual body motion, the proposed body state estimator indeed better captures the empirical robot motion.

The statistical summary of these five walks is listed in Table 1 and in Figure 8d, where root-mean-squared (RMS) errors are defined as the difference between the trajectories (II)–(IV) and the actual robot trajectory (I). Table 1 separately reports the means and standard deviations of the position RMSE errors of these five walks in the fore-aft and lateral directions. In contrast, Figure 8d individually plots the errors of the five walks, but mixing the RMS errors in fore-aft and lateral directions into one planar presentation. The figure shows that, in general, the nominal trajectory has the largest error, and the joint trajectory has a smaller error than the nominal one because it can catch the actual landing position on each step. Figure 8d also clearly shows that the proposed body state estimator has the best estimation of the empirical robot behavior. The IMU within the estimator catches the dynamics of the motion, and the configuration derived based on the joint configuration bounds the estimation at the trustable level, free from the notorious drift behavior when the estimator only contains state information from the IMU. In short, the proposed fusion strategy by EKF is effective for estimating the FP state in the current bipedal walking scenario. Furthermore, in Table 1, the small FP RMS errors of the estimated trajectory prove that the proposed body state estimator can derive a more accurate body state. Besides, the lateral motion of the robot during walking has a more dramatic state change than the fore-aft motion because the former has to perform oscillated FP shifting. This involves more dynamics, so the standard deviation on the lateral motion is larger than that on the fore-aft motion. However, as can be noticed in the figure, the shaking phenomenon occurred at the single-support stage, especially the touchdown moment. This indicates that the robot is unable to make full contact with the ground, mainly as a result of the backslash of the mechanism. Moreover, the estimated error is a result of the difference between the LIPM and the real robot. That is the reason why the body state feedback controller and foot contact corrector were developed for stabilizing the locomotion.

**Table 1:** RMS errors of the computed body trajectories (*i.e.*, FP) to the physical robot’s trajectory.

FP	Fore-Aft		Lateral	
	Mean	Std.	Mean	Std.
Nominal trajectory	6.78 mm	1.30 mm	7.04 mm	0.36 mm
Joint trajectory	5.17 mm	0.71 mm	7.05 mm	0.33 mm
Estimated trajectory	4.38 mm	0.65 mm	6.49 mm	0.43 mm

### 5.2. Evaluation of the Body State Feedback Control

To evaluate the performance of the control strategy, the robot with the closed-loop body position control implemented was set to walk within GTMS 5 times in the same scenario as the previous experiments (hereafter referred to as “closed-loop”). In these experiments, the robot also started from a standing state then walked 30 cm at 0.1 m/s on the force plate and returned to the standing posture. In addition, the robot operated with joint trajectories accordingly to the nominal FP trajectory generated by the open-loop preview controller, which was also carried out 5 times for comparison purposes (referred to as “open-loop”). Based on the experimental performance of the robot, the parameters of the controller $\left(k_{d},T_{d}\right)$ shown in Equation (6) are (0.92, 0.005) in the fore-aft direction and (0.82, 0.005) in the lateral direction. Experimental results show that the adequate range of $k_{d}$ for stable biped walking is around 0.5 to 1, and the adequate range of $T_{d}$ is no more than 0.01.

**Figure 9:**
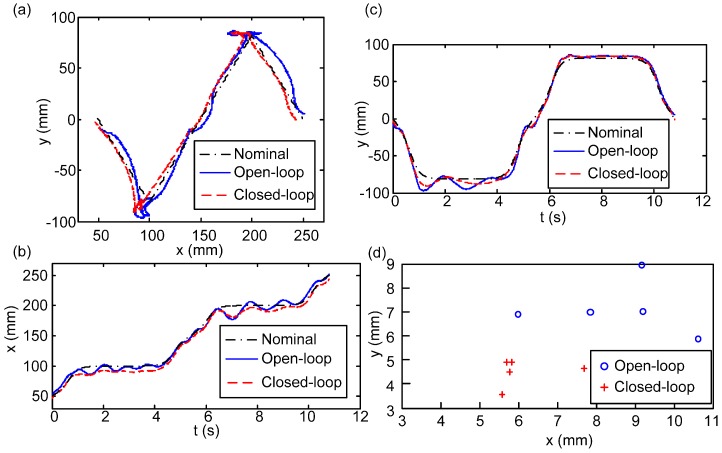
The robot body trajectories (*i.e.*, FP) derived by different methods: nominal trajectory (black dash-dotted), empirical robot trajectory with open-loop control (blue solid), and empirical robot trajectory with closed-loop control (red dashed). (**a**) Trajectories plotted in the xy-plane; (**b**) Fore-aft trajectory (x) *vs.* time; (**c**) Lateral trajectory (y) *vs.* time; (**d**) RMS errors of all walks.

One of the experiment results is plotted in Figure 9a–c as the exemplary presentation. The black, blue, and red lines represent the nominal trajectory, the robot motion generated according to the open-loop method, and the robot motion generated according to the closed-loop FP regulation method, respectively. Similar to the arrangement shown in Figure 8, subplot (a) shows the planar trajectory from the top view and subplots (b)–(c) plot the FP *vs.* time in the fore-aft (x) and lateral (y) directions. Though the robot with the closed-loop control strategy will still have some motion variation in comparison to the planned nominal one owing to the unmodeled ground contact/impact and non-rigid body structure, the robot with the closed-loop controller indeed exhibits less vibration and tracking errors in comparison to the robot programmed with the open-loop trajectory.

The statistical summary of these five walks in the closed-loop and five walks in the open-loop is detailed in Table 2 and in Figure 9d, where root-mean-squared (RMS) errors between the empirical robot trajectories (including open-loop or closed-loop) to the nominal trajectory are reported. Table 2 separately reports the means and standard deviations of the FP/ZMP RMSE errors of these five walks in the fore-aft (x) and lateral (y) directions. Similar to the style shown in Figure 8d, Figure 9d plots the errors of each walk but combines the RMS errors in the fore-aft and lateral directions into one planar presentation. The table and figure clearly show that the means and standard deviations of the RMS errors of both FP and ZMP are smaller when the robot has closed-loop control, so the robot with the closed-loop controller has better and consistent tracking performance to the nominal trajectories. The small ZMP RMS errors further indicate that the motion of the robot can fall into a more stable region as pre-planned.

**Table 2:** RMS errors of the robot body and ZMP trajectories to their nominal trajectories.

**FP**	**Fore-Aft**		**Lateral**	
	**Avg.**	**Std.**	**Avg.**	**Std.**
Open-loop	8.57 mm	1.39 mm	7.15 mm	1.12 mm
Closed-loop	6.62 mm	0.71 mm	4.52 mm	0.56 mm
**ZMP**	**Fore-Aft**		**Lateral**	
	**Avg.**	**Std.**	**Avg.**	**Std.**
Open-loop	26.59 mm	1.25 mm	22.42 mm	3.03 mm
Closed-loop	23.30 mm	1.02 mm	17.79 mm	2.04 mm

### 5.3. Evaluation of the Foot Posture Corrector

**Figure 10:**
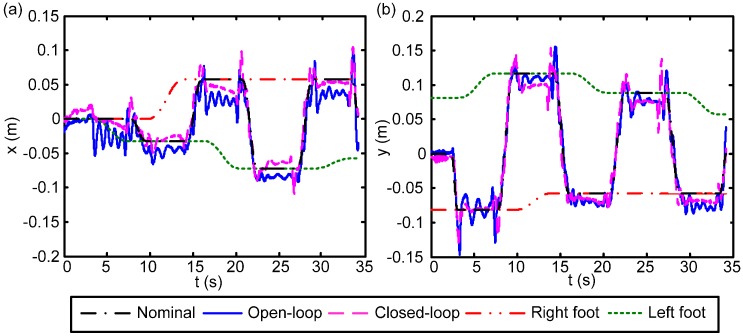
ZMP trajectories derived by different methods: nominal trajectory (black), empirical robot trajectory with open-loop control (blue), and empirical robot trajectory with closed-loop control (pink). (**a**) Fore-aft trajectory (x) *vs.* time; (**b**) Lateral trajectory (y) *vs.* time.

To evaluate the performance of the foot posture corrector, the robot with the proposed foot posture corrector implemented together with the body state feedback control was set to complete a 45° turning task. It is necessary to mention here that because the two controllers (*i.e.*, body state feedback controller and foot posture corrector) have different purposes, we did not intend for the experiments to be comparable but rather aimed at obtaining the correct setting for evaluation. The body state feedback is mainly for motion regulation, so the FP trajectory of a complete walking stride is provided. The traveling distance of one stride is already close to the length of the field of view of the vision system, and the force plate has a limited sensing area as well. In contrast, the second controller is mainly for foot posture correction. Because the foot tilting error of the robot was worse in turning than in straight walking, the in-place turning experiment was executed to evaluate the performance of the controller. The parameter setting and experimental scenario are similar to those described in [32], which reported on the turning of the robot. Additionally, the parameters relating to the foot posture corrector $\left(k_{d,torque},T_{d,torque}\right)$ shown in Equation (10) are (0.07, 0.5) based on the experimental performance of the robot. The experiment results of the ZMP trajectories are shown in Figure 10. The black, blue, and pink lines represent the nominal trajectory, the robot motion generated according to the open-loop method, and the robot motion generated accordingly to the closed-loop body regulation method with the proposed foot posture corrector, respectively. Similar to the arrangement illustrated previously, subplots (a) and (b) plot the ZMP *vs.* time in the fore-aft (x) and lateral (y) directions respectively. Note that here the ZMP data were collected by the force sensors mounted on the robot ankles according to Equation (7).

As shown in Figure 10, though the robot embedded with both the open-loop or closed-loop control can execute the planned turning task, the robot with the open-loop controller suffered greater disturbance owing to the unmodeled dynamics. Furthermore, it is visible that the open-loop robot’s performance involved more vibration in comparison with the closed-loop performance during experiments. As can be noted from Figure 10, during single-support conditions, the robot with the open-loop controller implemented experienced large vibrations while the robot with the closed-loop torque controller implemented was able to restrain such shaking, which further improved the stability of the turning behavior. Though there was still some oscillating behavior, the feet had better contact with the ground. The rubber pads installed at the bottom of the feet contribute to this unwanted behavior, even though they were intended to provide sufficient friction force and reduce the contact impact to protect the force/torque sensors installed at the ankle.

The statistical summary of these turning tasks is shown in Table 3 with open-loop and closed-loop controls, where the ZMP RMS errors in the fore-aft (x) and lateral (y) directions are listed. Apparently, the ZMP RMS are smaller when the robot has closed-loop control, which indicates that the motion of the robot can fall into a more stable region as pre-planned. Moreover, considering the fore-aft side, the foot posture corrector made a tremendous difference in reducing the ZMP RMS errors.

**Table 3:** RMS errors of the robot ZMP trajectories to the nominal ZMP trajectory.

ZMP	Fore-aft	Lateral
	Std.	Std.
Open-loop	21.1 mm	14.9 mm
Closed-loop	12.1 mm	14.1 mm

In order to test the stability and reliability of the proposed complete feedback control strategy (*i.e.*, body state feedback controller plus foot posture corrector), a relatively long and complex walk (147.5 s in total) was carried out to prove that the controller can be employed. In this task, the robot went forward first, then turned 90° left, and then turned 45° right. The task was successfully carried out and the shaking phenomenon caused by uneven contact with the ground can be reduced by employing the foot posture corrector. In addition, the RMS errors of the robot’s ZMP trajectories relative to the nominal ZMP trajectories are 18.7 mm and 19.3 mm in the fore-aft and lateral directions respectively.

## 6. Conclusions

In this paper, a sensor data fusion algorithm was developed to obtain the correct body motion for a bipedal robot during walking. In order to lower the measurement error and filter the signal noise, an EKF-based body state estimator was designed with sensory information from the joint encoders, the IMU, and the inclinometer. By employing this body state estimator, the true body state of the humanoid robot can be acquired, including translation at a specific fixed position (*i.e*., FP) on the body and orientation. Later, the proposed body state estimator was employed as a feedback control strategy to further improve the stability of the bipedal walking motion by regulating its body motion in real time. In addition, a foot posture corrector was introduced to reduce the extraneous torque resulting from the complex contact conditions between the robot’s soles and the ground. The performance of the estimator and the control strategy are assessed under the ground truth measurement system. Though there are some imperfections and disturbances, the tracking error arises from the discrepancy between the LIPM-based preview control and the real robot system. However, the proposed controller can still achieve some improvements compared with the open-loop strategy. First, the performance of the proposed body state estimator was evaluated by comparing its estimated FP trajectory with the empirical robot’s FP trajectory. The averaged RMS errors are 4.38 mm in the fore-aft direction and 6.49 mm in the lateral direction, which show improvements of 35% and 8%, respectively, compared to the nominal trajectory. Second, the body state feedback control algorithm is employed to regulate the actual FP position relative to the desired one. The performance of the controller is evaluated by the RMS errors of the robot’s FP and ZMP trajectories to the nominal FP and ZMP trajectories. With the proposed body state feedback control structure, the averaged RMS errors of the FP position improve 23% and 37%, respectively, in comparison with the robot operated by the open-loop method. This indicates that the proposed body regulation strategy is functional and stabilizes the robot’s walking motion. Finally, the proposed foot posture corrector was added to the system. During a turning task, the averaged RMS errors of the ZMP positions improve 42% and 5% respectively in comparison with the robot operated by the open-loop method.

We are in the process of developing a whole-body controller to obtain a more accurate CoM state by fusing force information and by using multiple models to cover the dynamics of the robot. We also plan to improve the control structure, which could then adjust the walking pattern in real time to realize reliable walking suitable for a wider range of environmental settings. At the same time, it would be feasible to carry out theoretical work on a combined controller which simultaneously takes the CoM and ZMP trajectories into account.

## Appendix

$$\begin{array}{l} H_{11}=\frac{2\left(q_{0}R_{23}+q_{1}R_{33}\right)}{R_{23}^{2}+R_{33}^{2}},H_{12}=\frac{2\left(-q_{1}R_{23}+q_{0}R_{33}\right)}{R_{23}^{2}+R_{33}^{2}}\\ H_{13}=\frac{2\left(-q_{23}R_{23}-q_{3}R_{33}\right)}{R_{23}^{2}+R_{33}^{2}},H_{14}=\frac{2\left(q_{3}R_{23}-q_{2}R_{33}\right)}{R_{23}^{2}+R_{33}^{2}}\\ H_{21}=\frac{2\left(-q_{0}R_{13}+q_{2}R_{33}\right)}{R_{13}^{2}+R_{33}^{2}},H_{22}=\frac{2\left(q_{1}R_{13}+q_{3}R_{33}\right)}{R_{13}^{2}+R_{33}^{2}}\\ H_{23}=\frac{2\left(q_{2}R_{13}+q_{0}R_{33}\right)}{R_{13}^{2}+R_{33}^{2}},H_{24}=\frac{2\left(-q_{3}R_{13}+q_{1}R_{33}\right)}{R_{13}^{2}+R_{33}^{2}}\\ H_{31}=\frac{2\left(q_{33}R_{11}-q_{0}R_{21}\right)}{R_{11}^{2}+R_{21}^{2}},H_{32}=\frac{2\left(q_{2}R_{11}-q_{1}R_{21}\right)}{R_{11}^{2}+R_{21}^{2}}\\ H_{33}=\frac{2\left(q_{1}R_{11}+q_{2}R_{21}\right)}{R_{11}^{2}+R_{21}^{2}},H_{34}=\frac{2\left(q_{0}R_{11}+q_{3}R_{21}\right)}{R_{11}^{2}+R_{21}^{2}} \end{array}$$

R represents the rotation matrix of the body frame {B} relative to the world frame {W}, which is defined in quaternion form as:

$$\begin{array}{l} R=\left[\begin{array}{ccc} R_{11} & R_{12} & R_{13}\\ R_{21} & R_{22} & R_{23}\\ R_{31} & R_{32} & R_{33} \end{array}\right]\\ =\left[\begin{array}{ccc} q_{0}^{2}+q_{1}^{2}-q_{2}^{2}-q_{3}^{2} & 2(q_{1}q_{2}-q_{0}q_{3}) & 2(q_{1}q_{3}+q_{0}q_{2})\\ 2(q_{1}q_{2}+q_{0}q_{3}) & q_{0}^{2}-q_{1}^{2}+q_{2}^{2}+q_{3}^{2} & 2(q_{2}q_{3}-q_{0}q_{1})\\ 2(q_{1}q_{3}-q_{0}q_{3}) & 2(q_{2}q_{3}+q_{0}q_{1}) & q_{0}^{2}-q_{1}^{2}-q_{2}^{2}+q_{3}^{2} \end{array}\right] \end{array}$$
